# Coping With Women’s Menstruation-Related Health Issues in Male-Dominated Companies: A Cross-Sectional Study in Japan

**DOI:** 10.7759/cureus.49569

**Published:** 2023-11-28

**Authors:** Yuan Li, Hiromi Kawasaki, Zhengai Cui, Satoko Yamasaki, Sae Nakaoka, Misaki Shiraishi

**Affiliations:** 1 Department of Health Science, Graduate School of Biomedical and Health Sciences, Hiroshima University, Hiroshima, JPN; 2 Department of Epidemiology and Public Health, School of Humanities and Management, Guangdong Medical University, Dongguan, CHN

**Keywords:** women's active participation, health behaviors, menstruation, female employees, women’s health, health literacy

## Abstract

Background and objectives: Japanese women face many female-specific health problems in the workplace, especially menstruation-related issues, which can adversely affect their quality of life and productivity. This study aims to examine how female employees in a male-dominated company in Hiroshima, Japan, cope with menstruation-related health issues in the workplace and the factors that influence their coping strategies.

Materials and methods: This study used a cross-sectional survey research method. The survey investigated age, health, and menstrual-related issues, as well as women's active participation support and health literacy (HL) levels. The sample population included employees of a manufacturer in Hiroshima prefecture. The data were collected from February 20 to March 10, 2023. The analysis subjects were 171 women who had experienced menstruation-related, women-specific health issues in the workplace. Their attitudes toward menstruation-related issues could influence their experiences. They were categorized into positive and negative groups based on their attitudes toward coping with women's health issues. Chi-square tests and logistic regression analysis were used to compare the two groups.

Results: The study found that 50.3% (N = 296) of female employees had experienced women's menstruation-related health issues at work. As many as 62.6% (N = 171) of female employees showed a positive attitude, and the study also found that female employees with a positive attitude toward menstruation-related health issues had higher HL (odds ratio (OR) = 1.216, 95% CI: 1.007-0.1.468) and were more likely to be able to predict menstruations (OR = 4.528, 95% CI: 1.618-12.670).

Conclusions: In male-dominated companies, many female employees are affected by menstruation-related problems in the workplace. A positive attitude toward women's health issues was positively associated with HL and predictive ability. Male-dominated companies should pay attention to women's menstruation-related health issues in the workplace, implement interventions to improve their health literacy and predictive ability, strengthen communication with them, and encourage them to actively participate in women's health initiatives.

## Introduction

Due to Japan's declining birth rate and aging population, the workforce is in sharp decline, and the Japanese government is encouraging more women to join the workforce. Compared with men, Japanese women face many female-specific health problems in the workplace. Most of these health problems are related to menstrual disorders and related symptoms. These mainly include a variety of menstrual dysfunctions (e.g., cycle disorders, excessive menstruation), gynecological diseases/syndromes (e.g., endometriosis, polycystic ovary syndrome), menstruation-related symptoms (e.g., premenstrual syndrome (PMS), menopausal symptoms), reproductive outcomes (e.g., infertility, duration of pregnancy), and biological outcomes (e.g., serum sex hormones)) [1]. The most common of these relate to painful menstruation (also known as dysmenorrhea), heavy menstrual bleeding (HMB), and PMS [2]. It has been reported that 80% of Japanese women experience dysmenorrhea [3]; nearly 20% of Japanese women experience HMB [4]; and more than 25% of Japanese women of reproductive-age experience PMS [5]. Menstruation-related issues increase women's psychological stress, which can lead to mental health problems such as anxiety and depression. Menstruation-related problems can adversely affect women's quality of life and productivity, resulting in substantial economic and social burdens [2,4,6].

How Japanese women cope with menstruation-related health problems in the workplace is an issue. Studies have shown that how they cope with health problems is closely related to their level of health literacy (HL) [7,8]. The World Health Organization (WHO) defines HL as “cognitive and social skills that determine an individual's motivation and ability to acquire, understand, and use information to promote and maintain physical health” [9]. Studies have also found that high HL can improve workers' health, while low HL can limit workers' understanding of occupational health and safety [10].

At the same time, the way they cope with health problems can be influenced by their sociocultural and work-related environments. It has been reported that Japanese women experience more negative emotions during menstruation than women in other countries [11]. A research study comparing Japan and Taiwan showed that there is a lack of menstrual education both within Japan and in the wider society, and related to this is a lack of menstrual information sharing between Japanese parents and children [12]. Most Japanese women find it natural to experience menstrual pain, which somehow influences how they cope with it [5].

Compared to men, Japanese women face more barriers to employment and work in the workplace. Female employees have specific health and safety requirements that differ from their male counterparts. In general, women are more likely to report work-related health problems and may be more susceptible to health issues such as menstruation-related problems, especially in male-dominated professional environments [13]. Previous studies have shown that women face more gender inequality in male-dominated corporate work environments [14]. A male-dominated company is an organization where men comprise the majority of employees, particularly in managerial positions [15]. How women-specific health issues such as menstruation are perceived and managed can have a direct impact on women's health and productivity.

However, research on how women in male-dominated companies cope with menstruation-related health issues is limited in Japan. For this reason, this study aims to examine the factors that influence how female employees in a male-dominated company in Hiroshima, Japan, cope with women-specific health issues such as menstruation in the workplace. The findings of this study will inform the company's decision-making to promote better participation of female employees in the workplace and to promote women's health.

## Materials and methods

Study design

A cross-sectional survey design was used in this study.

Survey instruments

This questionnaire comprised self-defined questions regarding participants' age, position, health, and menstrual-related issues (Table 1) [7,16].

**Table 1:** Questionnaire PMS: premenstrual syndrome

Questions	Options
Age (in years)	20s
	30s
	40s
	50s-60s
Are you a manager?	Yes
	No
Cancer screening for women	Screened for breast and cervical cancer
	Only screened for breast cancer
	Only screened for cervical cancer
	Neither
Are you aware that female hormones can cause irregular periods, menstrual cramps, and PMS?	I know
	I have heard of it
	I do not know
	I have never heard of it
Have you ever experienced problems due to health issues specific to women or symptoms that are more common among women in the workplace?	Yes
	No
Can you deal with painful symptoms in a positive way during menstruation?	Quite capable
	Can
	Cannot
	Not at all
Can you predict menstruation based on changes in your physical condition?	Quite capable
	Can
	Cannot
	Not at all
Are you aware of the physical and mental changes associated with menstruation?	Quite capable
	Can
	Cannot
	Not at all
Symptoms you experienced as a problem at your place of employment *Multiple answers are acceptable	Menstruation-related symptoms and diseases
	PMS
	Cancers common in women
	Menopause
	Mental health
	Infertility/fertility
	None applicable
	Others
Learn about the company's women's active participation support	I know
	I have heard of it
	I do not know
	I have never heard of it

Additionally, it included a health literacy questionnaire (Table 2) [17].

**Table 2:** Health literacy scale The higher the score, the higher the health literacy level. The internal consistency of the scale used in this study was sufficient (Cronbach's α=0.818).

Questions	Options
Can you extract relevant information about women's health from various sources?	Four-point Likert scale (4=Quite capable, 3=Can, 2=Cannot, 1=Not at all)
Can you evaluate the credibility of information about women's health?	
Can you ask clarifying questions when you encounter unfamiliar medical terminology or concepts?	
Can you gather information about women's health topics upon request?	
Can you comprehend information about women's health that you encounter in daily life?	

The survey instruments were all in the local language, Japanese.

Sample and data collection

A manufacturer in Hiroshima prefecture conducted a survey to promote women's empowerment. The analysis leveraged the data from the survey, with the intention of implementing measures to promote women's active participation. The company, a large gas producer and manufacturing company, had a large number of male employees and was male-dominated. The company's products had the highest sales volume in Hiroshima prefecture and actively engaged in sustainable development goals (SDGs) such as carbon dioxide (CO_2_) reduction and carbon neutrality.

The sample population was all employees of the company. The company had a total of 1,416 employees, of which 1,042 were male and 374 were female. The data collection period was from February 20 to March 10, 2023. This study used secondary data from a survey conducted at the company in Hiroshima, Japan.

Ethical consideration

The studies involving humans were approved by the Institutional Review Board, Hiroshima University Epidemiology Ethics Committee, Hiroshima, Japan (approval no. E2023-0102). The studies were conducted in accordance with local legislation and institutional requirements.

As part of the training aimed at promoting female employees' active participation, a questionnaire was administered to all employees by the company. The questionnaire consisted of questions that the company believes are necessary to address the SDGs and the challenges for women's advancement. Responses were voluntary and did not include names. Responses were deemed consent to the survey. After a basic analysis was performed by the healthcare personnel, the data were provided to Hiroshima University, Hiroshima, Japan, for detailed analysis with the consent of the administrator.

Data analysis 

Participants were categorized into positive and negative groups based on attitudes toward pain during menstruation. We manipulatively defined the response that they actively coped with menstruation as a positive attitude toward women's health issues. 

**Figure 1:**
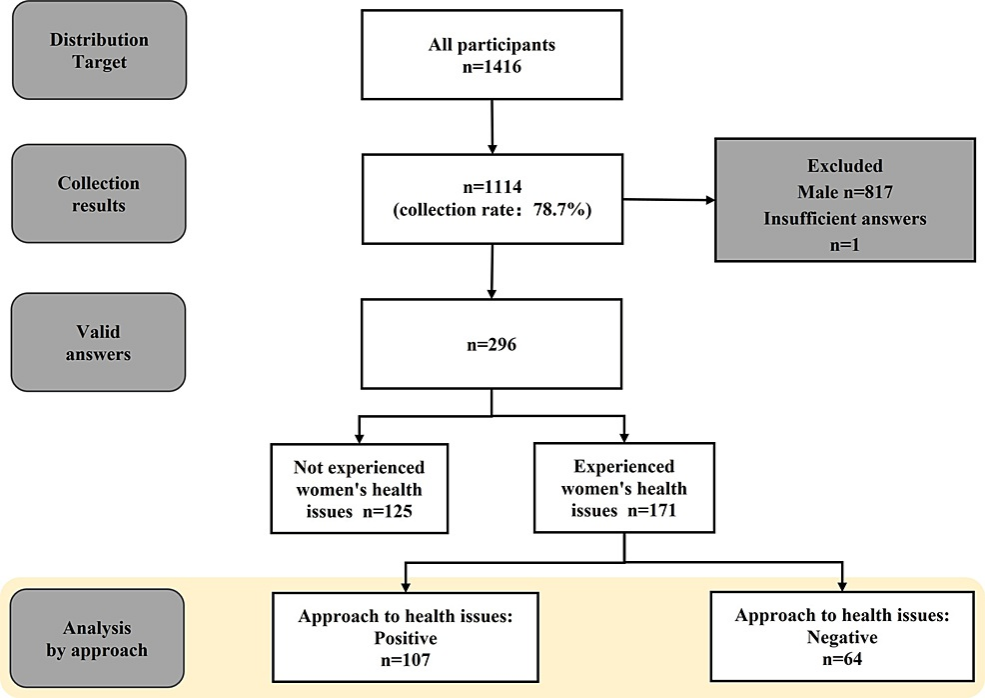
A flowchart of respondents' responses and analysis by attitude toward women's health issues

The analysis targets are shown in Figure 1.

Of the 1,416 distribution targets, a total of 1,114 responses (78.7%) were received. Of these respondents, 817 were males, and one response was insufficient. Therefore, 296 respondents (20.9%, N = 1416) were included in the current analysis. First, respondents were divided into two groups: those who had not experienced women's health issues (n = 125, 42.2%, N = 296) and those who had experienced women's health issues (n = 171, 57.8%, N = 296) in the workplace.

Further, 171 people who had experiences where women's health was an issue at work were included in the analysis to examine the factors for attitudes that influence how female employees in a male-dominated company with women-specific health issues such as menstruation function in the workplace (Figure 1).

The respondents who experienced women's health issues in the workplace were classified into positive (n = 107, 62.6%) and negative (n = 64, 37.4%) groups.

This study used the following analytical techniques: First, descriptive statistics were used to summarize data on demographic and women's health issues. Then, chi-square tests were used to compare and investigate differences in demographic characteristics, women's menstruation-related health issues, women's active participation support, and HL between the positive and negative groups. Finally, logistic regression analysis was performed with the factors that were significant in the chi-square tests as independent variables and the positive/negative groups (“whether they have a positive attitude toward women's health issues”) as dependent variables.

For data analysis, IBM SPSS software version 28 (IBM Corp., Armonk, NY) was used to calculate the percentage (%), mean, and standard deviation (SD) to describe the data statistically. The results of logistic regression were presented as odds ratios (ORs) and 95% confidence intervals (CIs). A p-value <0.05 was considered statistically significant.

## Results

Participants' characteristics

According to statistics, the company had a total of 1,416 employees, consisting of 1,042 male employees (73.6%) and 374 female employees (26.4%). The male-to-female ratio was 2.79 (1,042/374). The company had a total of 349 managers, consisting of 296 male managers (84.8%) and 53 female managers (15.2%). The male-to-female ratio in management was 5.58 (296/53), approximately twice the company's overall male-to-female ratio. Moreover, it was evident that male managers dominated most of the company's decision-making processes. Therefore, the company can be characterized as a typically male-dominated organization.

In this study, the analysis population was 171 subjects, with 26 (15.2%) female managers and 145 (84.8%) general female employees, including 38 (22.2%) in their 20s, 30 (17.5%) in their 30s, 36 (21.1%) in their 40s, and 67 (39.2%) in their 50s or older.

Of the study participants (N = 171), 43 (25.1%) were screened for cervical and breast cancer, 12 (7.0%) were screened for breast cancer only, 60 (35.1%) were screened for cervical cancer only, and the remaining 56 (32.7%) did not receive any screenings.

The mean score for HL of the 171 subjects in this study was 14.12, with a standard deviation of 1.89.

In addition, as far as women's health issues are concerned, of the 296 female employees who participated in the survey, 171 (57.8%, N = 296) of them had experienced women's health issues in the workplace. The main problems they faced were related to menstrual problems, with a whopping 87.1% (N = 171) experiencing these troubles. In other words, 50.3% (N = 296) of female employees experienced menstrual-related health problems in the workplace. For example, 141 (82.5%, N = 171) of them reported symptoms and diseases related to menstruation (irregular menstruation, painful menstruation, etc.), and 66 (38.6%, N = 171) of them reported PMS (Table 3).

**Table 3:** Participants' characteristics Data are presented as numbers and percentages (%) except for health literacy which is presented as mean and standard deviation (SD). PMS: premenstrual syndrome

Characteristics	n	%
Age (years)		
20s	38	22.2
30s	30	17.5
40s	36	21.1
50s-60s	67	39.2
Are you a manager?		
Yes	26	15.2
No	145	84.8
Cancer screening for women		
Screened for breast and cervical cancer	43	25.1
Only screened for breast cancer	12	7.0
Only screened for cervical cancer	60	35.1
Neither	56	32.7
Are you aware that female hormones can cause irregular periods, menstrual cramps, and PMS?		
I know	170	99.4
I have heard of it		
I do not know	1	0.6
I have never heard of it		
Have you ever experienced problems due to health issues specific to women or symptoms that are more common among women in the workplace?		
Yes	171	100.0
No		
Can you deal with painful symptoms in a positive way during menstruation?		
Quite capable	4	2.3
Can	103	60.2
Cannot	59	34.5
Not at all	5	2.9
Can you predict menstruation based on changes in your physical condition?		
Quite capable	20	11.7
Can	118	69.0
Cannot	29	17.0
Not at all	4	2.3
Are you aware of the physical and mental changes associated with menstruation?		
Quite capable	16	9.4
Can	133	77.8
Cannot	22	12.9
Not at all		
Symptoms you experienced as a problem at your place of employment *Multiple answers are acceptable		
Menstruation-related symptoms and diseases	141	82.5
PMS	66	38.6
Cancers common in women	6	3.5
Menopause	39	22.8
Mental health	20	11.7
Infertility/fertility	11	6.4
None applicable	1	0.6
Others	7	4.1
Learn about the company's women's active participation support		
I know	39	22.8
I have heard of it	45	26.3
I do not know	84	49.1
I have never heard of it	3	1.8
Health literacy (mean ± SD)	14.12	±1.89

Correlation between participants' characteristics and attitudes toward women's health issues

The chi-square test results showed significant differences between the positive and negative groups in terms of HL (p =0.045), as well as the following questions: Can you predict menstruation based on changes in your physical condition? (p <0.001)? Are you aware of the physical and mental changes associated with menstruation (p = 0.002)? However, there were no significant differences in terms of age (p = 0.447), cervical and breast cancer screening status (p = 0.158), whether they knew that female hormones could cause irregular menstruation, dysmenorrhea, and PMS (p = 0.374), whether they knew about the company's women's active participation support (p = 0.27), or whether they were managers (p = 0.217) (Table 4).

**Table 4:** Correlation between participants' characteristics and attitudes toward women's health issues Data are presented as numbers and percentages (%) except for health literacy which is presented as mean and standard deviation (SD). * p <0.05 (significant) PMS: premenstrual syndrome

Characteristics	Attitudes toward women's health issues		p-value
	Positive	Negative	
	107(62.6%)	64(37.4%)	
Age (years)			
20s	24 (63.2)	14 (36.8)	0.447
30s	15 (50.0)	15 (50.0)	
40s	23 (63.9)	13 (36.1)	
50s-60s	45 (67.2)	22 (32.8)	
Cancer screening for women			
Screened for breast and cervical cancer	33 (76.7)	10 (23.3)	0.158
Only screened for breast cancer	7 (58.3)	5 (41.7)	
Only screened for cervical cancer	36 (60.0)	24 (40.0)	
Neither	31 (55.4)	25 (44.6)	
Are you aware that female hormones can cause irregular periods, menstrual cramps, and PMS?			
Yes	107 (62.9)	63 (37.1)	0.374
No	0 (0.0)	1 (100.0)	
Learn about the company's women's active participation support			
Yes	55 (65.5)	29 (34.5)	0.27
No	52 (59.8)	35 (40.2)	
Health literacy (mean ± SD)	14.43±1.64	13.59±2.16	0.045*
Can you predict menstruation based on changes in your physical condition?			
Yes	97 (70.3)	41 (29.7)	<0.001*
No	10 (30.3)	23 (69.7)	
Are you aware of the physical and mental changes associated with menstruation?			
Yes	100 (67.1)	49 (32.9)	0.002*
No	7 (31.8)	15 (68.2)	
Are you a manager?			
Yes	14 (53.8)	12 (46.2)	0.217
No	93 (64.1)	52 (35.9)	

Associated factors for attitudes toward women's health issues

The results of the logistic regression analysis showed that those participants with positive attitudes toward women's health issues such as menstruation had higher HL (OR = 1.216, 95% CI: 1.007-0.1.468) and were more likely to be able to predict menstrual problems based on changes in physical conditions (OR =4.528, 95% CI: 1.618-12.670) than people with negative attitudes (Table 5).

**Table 5:** Associated factors for attitudes toward women's health issues * p <0.05 (significant) OR: odds ratio

Variables	Negative vs. Positive	
	OR (95% CI)	p-value
Health literacy	1.216 (1.007-1.468)	0.042*
Can you predict menstruation based on changes in your physical condition?		
No	ref.	
Yes	4.528 (1.618-12.670)	0.004*
Are you aware of the physical and mental changes associated with menstruation?		
No	ref.	
Yes	1.159 (0.323-4.154)	0.821

## Discussion

Firstly, to our knowledge, this is the first article to examine women's health issues in male-dominated Japanese companies. This study shows that up to 50.3% (N = 296) of female employees experience menstruation-related health problems at work. This is consistent with previous studies [3,5]. However, this figure is likely to be an underestimation. A possible explanation is that menstruation is relatively taboo in Japan, and female employees cannot freely talk about issues related to the menstrual cycle with management, so they naturally cannot obtain support from management [6,16]. Similarly, men in male-dominated occupational environments are reluctant to discuss mental health issues [18]. This is not to mention their lack of concern for female employees. In addition, previous studies have shown that women in manufacturing, a traditionally male-dominated industry, are highly exposed to health and safety hazards, such as poor working conditions, unfair labor practices, low wages, shift work, and night work, which can lead to an increased risk of spontaneous abortion among women. This exposure also puts them at greater risk for adverse health and mental threats and makes them less likely to receive adequate health care [19-21]. Therefore, companies should pay attention to the menstruation-related health issues of female employees in the workplace. They should establish effective and smooth communication channels so that female employees can have a rest without hesitation in the case of a non-sick condition, which is a feminine physiological problem.

Secondly, our study found that a positive attitude toward women's menstruation-related health issues was positively correlated with health literacy. Female employees with high health literacy are more likely to adopt positive coping attitudes for health problems such as menstruation in the workplace. This is consistent with previous research [17,22]. High health literacy is associated with positive health behaviors and attitudes, which can lead to improved job performance [7]. Health literacy is an essential component of women's ability to understand, process, and act on health-related information. It encompasses both health promotion and disease prevention activities [23]. Therefore, improving the health literacy of female employees can encourage them to adopt a more positive attitude towards menstruation-related health issues. The implementation of female employees' active participation support is an effective opportunity to enhance the health literacy of female employees. These interventions include the development of health education materials, clear and effective communication, and education to increase health literacy, self-efficacy, and self-advocacy skills [8].

Thirdly, to our knowledge, this study is the first to directly examine the relationship between health behaviors and individual predictive ability. Our findings suggest that positive attitudes toward women's menstruation-related health issues were positively associated with health predictive ability, such that employees with higher predictive ability were more likely to adopt positive behaviors to cope with women's health issues, such as menstruation, that exist in the workplace. Although there is currently a lack of directly relevant research, theories such as the Theory of Planned Behavior, the Health Belief Model, and Self-Efficacy provide possible explanatory perspectives [24-26]. That is, when individuals have the necessary predictive abilities, they can perceive the risks and possible consequences of menstruation-related health problems through the changes in their bodies and adjust work plans and divisions in advance to inconveniences. They are also more likely to seek out health information and learn about the risks and benefits of different health behaviors. This leads to higher motivation to adopt positive coping attitudes and strategies [27]. The ability to predict the future is directly related to the amount of information an individual has. The more valid and relevant information people have, the more accurate their predictions will be, and the more likely they are to take positive actions to achieve their goals [28]. Effective communication can improve women's ability to understand and retain information [29].

This study also has some limitations. A questionnaire survey by the company in Hiroshima prefecture that is engaged in support aimed at promoting female employees' active participation was used secondarily. The question items were limited, so the possibility of selection bias exists. Further studies are needed to explore the generalizability of our findings, particularly with regard to examining the explicit perceptions of male employees in male-dominated companies about women's health issues. Furthermore, understanding how women in male-dominated occupations manage unique health challenges is crucial for fostering greater female participation in the workplace. This is a significant topic to explore, especially when compared to female-dominated or gender-balanced occupations.

## Conclusions

In male-dominated companies, female employees face more complex work environments and are often affected by menstruation-related and other health issues. Research has shown that positive coping attitudes are positively associated with health literacy and predictive ability when faced with women's health issues.

Therefore, it is important for male-dominated companies to emphasize and address women-specific health issues by developing health education materials, clear and effective communication, and increased education on health literacy, self-efficacy, and self-advocacy skills. These will help to improve the health literacy and predictive skills of female employees, motivate them to positively cope with women's health issues in the workplace, and effectively promote women's active participation.
